# The Role of Dietary Habits, Night-Time Feeding and Oral Hygiene in Early Childhood Caries: A Retrospective Observational Study in 248 Children from Southern Italy

**DOI:** 10.3390/children13040489

**Published:** 2026-03-31

**Authors:** Luisa Limongelli, Vanja Granberg, Francesca Cervinara, Tommaso Corsalini, Daniela Di Venere, Ilaria Fricelli, Massimo Corsalini

**Affiliations:** Department of Interdisciplinary Medicine, University of Bari “Aldo Moro”, Piazza Giulio Cesare, 11, 70124 Bari, Italy; luisa.limongelli@uniba.it (L.L.); cervinarafrancesca@gmail.com (F.C.); tommasocorsalini@gmail.com (T.C.); daniela.divenere@uniba.it (D.D.V.); ilariafricelli@gmail.com (I.F.); massimo.corsalini@uniba.it (M.C.)

**Keywords:** early childhood caries, night-time feeding, sugar-rich diet, feeding duration, oral hygiene, fluoride toothpaste, pediatric dentistry, caries risk factors, prevention

## Abstract

**Highlights:**

**What are the main findings?**

- Prolonged night-time feeding beyond 12 months is associated with increased ECC risk, with a progressive increase observed with longer duration.
- A high-sugar diet, high daily meal frequency, and delayed initiation of toothbrushing are associated with higher ECC prevalence, whereas electric toothbrush and fluoridated toothpaste use are protective.

**What are the implications of the main findings?**

- ECC prevention should prioritize early cessation of night-time feeding and reduction in sugar exposure over focusing solely on feed type.
- Early-life dietary counseling combined with timely introduction of oral hygiene and fluoride use is essential to reduce ECC burden.

**Abstract:**

Background: Early Childhood Caries (ECC) is a prevalent multifactorial disease strongly influenced by dietary and behavioral factors. Night-time feeding practices and sugar exposure have been implicated, yet the relative impact of feeding duration, feeding type, and oral hygiene remains debated. This study aimed to investigate the association between ECC and major dietary and behavioral risk factors, with particular emphasis on the presence and duration of night-time feeding, in a pediatric population from Southern Italy. Methods: A single-center retrospective observational study was conducted on medical records of children aged 1–6 years referred for a first dental visit to a pediatric dentistry unit. ECC and severe ECC (S-ECC) were diagnosed according to AAPD criteria. Data on night-time feeding (presence and duration), sugar-rich diet, number of daily meals, oral hygiene habits, and age at initiation of toothbrushing were collected through structured interviews. Associations were evaluated using chi-square tests and multivariable logistic regression analysis, with ECC/S-ECC as the dependent variable. Results: A total of 248 children were included. ECC/S-ECC prevalence was 62.5%. A sugar-rich diet was associated with increased ECC risk (OR = 4.14, *p* < 0.001). Prolonged night-time feeding showed a dose–response relationship with ECC, with risk increasing beyond 12 months and exceeding twelvefold for durations > 24 months. Multivariable analysis showed that night-time feeding duration > 12 months, high-sugar diet, >5 daily meals, and delayed initiation of toothbrushing were associated with ECC, whereas use of an electric toothbrush and fluoridated toothpaste showed a trend toward a protective effect. Feeding type was not independently associated with ECC after adjustment for duration. Conclusions: Duration of night-time feeding, rather than feeding type, represents a key modifiable determinant of ECC risk. Preventive strategies should prioritize early cessation of night-time feeding, reduction in sugar exposure, limitation of meal frequency, and early introduction of effective oral hygiene with fluoride.

## 1. Introduction

Early Childhood Caries (ECC) is defined as the presence of one or more decayed (cavitated or non-cavitated), missing, or filled surfaces in any primary tooth in a preschool child between birth and 71 months of age. In children younger than 3 years, the presence of any carious lesion on smooth surfaces is diagnostic of Severe Early Childhood Caries (S-ECC). In children aged 3 to 5 years, S-ECC is defined by age-specific dmft (decayed, missing, filled teeth) thresholds (≥4 at 3 years, ≥5 at 4 years, and ≥6 at 5 years) and/or by the presence of smooth-surface carious lesions affecting the maxillary primary anterior teeth [1].

Recent systematic reviews and meta-analyses indicate that Early Childhood Caries remains a highly prevalent condition worldwide, affecting approximately 48–49% of preschool children. Despite advances in preventive strategies, the global burden of ECC has not significantly declined and may even show a slight increasing trend in recent years.

**Figure 1:**
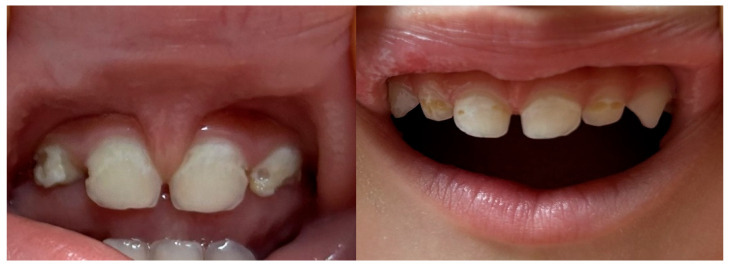
Clinical photographs of early ECC lesions in patients aged 1.6 and 2.4 years.

The distribution of ECC remains highly heterogeneous across regions, with higher prevalence reported in low- and middle-income countries and among socioeconomically disadvantaged populations. Overall, ECC continues to represent a major public health concern, affecting hundreds of millions of children worldwide and contributing substantially to disease burden in early life [2,3]. At the earliest stage of ECC, lesions commonly manifest as chalky white spot lesions or as a continuous white band involving the cervical third of the maxillary primary incisors (Figure 1).

**Figure 2:**
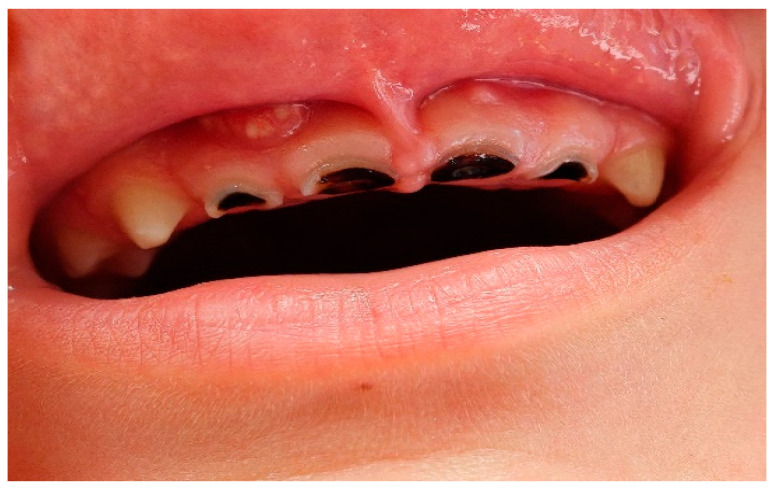
Clinical photograph of Severe Early Childhood Caries (S-ECC) in a 3.6-year-old patient.

Initial lesions typically develop along the gingival margin and on interproximal or palatal surfaces, and, in advanced presentations, may also involve the incisal edge [4,5]. As caries progresses, cavitation occurs and lesions may appear yellow, brown, or black. In severe cases, the dental crown may be extensively destroyed, leaving only the residual root structure visible (Figure 2).

Diagnosis is primarily based on careful visual clinical examination supported by a targeted dietary and behavioral history and, when indicated, by radiographic assessment. In particular, bitewing radiographs, when adequate patient cooperation is achievable, may assist in the detection of interproximal lesions and in the assessment of lesion extent [6].

Standardized criteria for the diagnosis and reporting of ECC were proposed following a workshop on early childhood caries in the late 1990s [7] (Table 1). This classification distinguishes early forms from severe forms based on the number and location of affected tooth surfaces and on the DMFS (decayed, missing, filled surfaces) score, using increasingly stringent criteria with increasing age.

In 1978, the American Academy of Pedodontics and the American Academy of Pediatrics issued a joint statement (‘Nursing Bottle Caries’) addressing severe forms of caries in young children associated with inappropriate bottle-feeding practices [1]. However, in the following decades, it became clear that inappropriate bottle-feeding was not the sole causal factor of this condition, and a multifactorial etiology was proposed.

In 1994, the Centers for Disease Control and Prevention (CDC) recommended the term *early childhood caries (ECC)* in order to emphasize the multiple factors contributing to the development of caries in young children. The etiology of ECC results from the interaction of microbiological, dietary, environmental, and socioeconomic factors [3], leading to an imbalance between enamel demineralization and remineralization processes.

From a microbiological perspective, ECC is strongly associated with early colonization by Streptococcus mutans and Streptococcus sobrinus [8], which are mainly transmitted from the mother or caregivers through salivary contact [9,10]. The high availability of fermentable carbohydrates, particularly sucrose, promotes the production of organic acids and the rapid progression of carious lesions.

Inappropriate feeding practices, such as prolonged bottle use [1,11,12], night-time feeding beyond the first year of life, and a high frequency of sugar intake, represent key risk factors. These factors are further exacerbated by the reduction in salivary flow during sleep. Environmental factors also play an important role, including poor oral hygiene, qualitative and quantitative alterations in saliva, and enamel defects. In addition, socioeconomic determinants—such as low parental educational level and limited access to dental care—contribute significantly to the development of the disease.

The management of ECC is based on an individualized caries risk assessment, which guides the selection of appropriate preventive and therapeutic strategies. In children at low risk, educational and behavioral interventions are recommended, whereas in those at moderate or high risk, preventive measures should be combined with targeted restorative treatments aimed at stabilizing lesions and controlling disease progression, taking into account the child’s age, clinical severity, and level of cooperation. Therapeutic approaches should prioritize minimally invasive and child-centered techniques, reserving more complex procedures, including sedation or general anesthesia, for selected cases.

The primary aim of this study was to investigate the main risk factors associated with Early Childhood Caries (ECC), with particular emphasis on the role of night-time feeding.

Additional analyses investigated the relationship between clinical caries severity and other behavioral factors, such as oral hygiene practices and the frequency of sugar intake. Collectively, these outcomes were considered to identify the main determinants of caries risk in the studied pediatric population and to evaluate the potential impact of night-time feeding habits on disease progression. The ultimate aim of this study is to expand and update current scientific evidence while providing practical tools for healthcare professionals and parents, who play a key role in the daily management of children’s feeding practices and oral hygiene.

## 2. Materials and Methods

### 2.1. Study Design

A single-center retrospective observational study based on the review of clinical records of children attending their first dental visit at the Giovanni XXIII Children’s Hospital in Bari.

The study was approved by the local Ethics Committee (prot. 2381/CEL) and conducted in accordance with the Declaration of Helsinki. Written informed consent was obtained from parents or legal guardians. All data were collected in anonymized form and used exclusively for scientific purposes.

Clinical records of all patients evaluated between September 2024 and September 2025 were retrospectively reviewed.

Children aged between 1 and 6 years attending their first dental visit at the Pediatric Dentistry Unit during the study period were eligible for inclusion.

Additional inclusion criteria were the presence of erupted primary dentition and the availability of complete clinical and anamnestic information in the medical record.

After clinical examination, children were classified according to the criteria of the American Academy of Pediatric Dentistry (AAPD) as having no early childhood caries (no ECC), early childhood caries (ECC), or severe early childhood caries (S-ECC).

**Figure 3:**
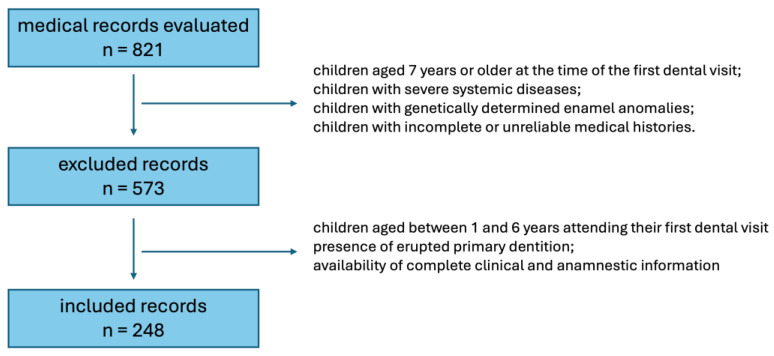
Flow diagram of medical record selection and inclusion in the study.

Exclusion criteria were: (i) severe systemic diseases potentially interfering with dental development; (ii) genetically determined structural enamel anomalies; (iii) incomplete or unreliable medical history (Figure 3).

### 2.2. Data Collection

As part of routine clinical care, parents complete a standardized medical and behavioral questionnaire at the first visit (Supplementary File S1).

This questionnaire includes information on dietary habits, feeding practices, oral hygiene behaviors, and other relevant anamnestic variables. Night-time feeding practices were recorded based on parental reports and included both the type of feeding and its duration. Due to the small number of observations in some categories, the variable describing the type of night-time feeding was recoded into two groups: breastfeeding (maternal milk) and other types of feeding.

The duration of night-time feeding was categorized according to parental reports, and a threshold of 12 months was used in the analysis.

Dietary sugar intake was evaluated based on the reported consumption of sugar-containing foods. A high-sugar diet was defined as the frequent intake of at least two sugar-rich foods within the same day.

Additional variables included the number of daily meals, the use of fluoridated toothpaste, and the age at initiation of toothbrushing.

A detailed distribution of all collected variables is provided in Supplementary Table S2.

### 2.3. Caries Risk Assessment

Dental examinations were performed by pediatric dentists through visual clinical inspection. The following were recorded: (i) cavitated and non-cavitated carious lesions; (ii) filled tooth surfaces; (iii) primary teeth missing due to caries.

The diagnosis of ECC and S-ECC was established according to AAPD criteria [1], taking into account patient age, lesion distribution, and involvement of smooth surfaces.

Patients were stratified into caries risk categories (low, moderate, high) based on a combination of: (i) clinical findings; (ii) behavioral factors; (iii) feeding habits; and (iv) reported socio-environmental conditions.

### 2.4. Statistical Analysis

Collected data were entered into an electronic database and analyzed using statistical software. Categorical variables were described as absolute frequencies and percentages. The sample was divided into two groups according to dental diagnosis: children with ECC/S-ECC and children without ECC.

Associations between diagnosis and dietary and behavioral variables—particularly type and duration of night-time feeding and presence of a sugar-rich diet—were evaluated. Owing to fragmentation of the original categories, the variable “type of night-time feeding” was recategorized as breastfeeding versus other types of feeding.

Associations between categorical variables were assessed using the Chi-square (χ^2^) test of independence.

All variables were selected a priori based on clinical relevance and existing literature on ECC risk factors. No automated variable selection procedures (e.g., stepwise methods) were applied.

A multivariable logistic regression model was fitted to estimate the association between ECC (dependent variable: 1 = ECC/S-ECC, 0 = no ECC) and selected dietary and behavioral predictors. The model was specified as follows:
*Logit (p) = β0 + β1 (toothbrushing > 1/day) + β2 (electric toothbrush) + β3 (fluoridated toothpaste) + β4 (high-sugar diet) + β5 (night-time feeding) + β6 (night-time feeding > 12 months) + β7 (>5 meals/day) + β8 (brushing start 1–2 years) + β9 (brushing start 2–3 years) + β10 (brushing start > 3 years)*

All variables were coded as binary indicators (0 = no, 1 = yes). The reference category for age at initiation of toothbrushing was <1 year. Only subjects with complete data for all variables included in the model were analyzed (complete-case analysis). The final sample consisted of 90 children (63 with ECC/S-ECC and 27 without ECC). Cases with missing data were excluded listwise from the multivariable analysis. AI-assisted tools were used for graphical visualization only. All analyses, data interpretation, and final verification of graphical outputs were performed by the authors.

## 3. Results

Out of a total of 821 medical records screened, 248 patients were included in the analysis. Within this defined sample, a high prevalence of ECC/S-ECC was observed, with heterogeneous distribution in relation to feeding and oral hygiene habits. Children affected by ECC more frequently exhibited night-time feeding persisting beyond 12 months of age and high exposure to sugar-rich diets.

### 3.1. Sugar-Rich Diet and ECC

The association between sugar consumption and ECC was statistically significant. After excluding cases with missing data, the final sample consisted of 207 children. Among children without a sugar-rich diet, 41.2% presented ECC (21/51), indicating that the disease may occur even in the absence of high sugar intake. However, in children with a sugar-rich diet, the prevalence increased to 74.4% (116/156).

The Chi-square test revealed a statistically significant association between a sugar-rich diet and ECC (χ^2^(1) = 17.46; *p* < 0.001), which was further supported by an odds ratio of 4.14 (95% CI: 2.13–8.04), indicating an approximately fourfold higher risk in children with high sugar consumption.

Overall, these findings reinforce existing evidence linking a sugar-rich diet to an increased prevalence of early childhood caries.

### 3.2. Duration of Night-Time Feeding

Duration of night-time feeding showed a strong association with the presence of ECC. Night-time feeding duration was categorized into four groups: no night-time feeding, duration < 12 months, duration between 12 and 24 months, and duration > 24 months. Only children with complete data regarding both dental diagnosis and night-time feeding duration were included in the analysis (Table 2).

**Figure 4:**
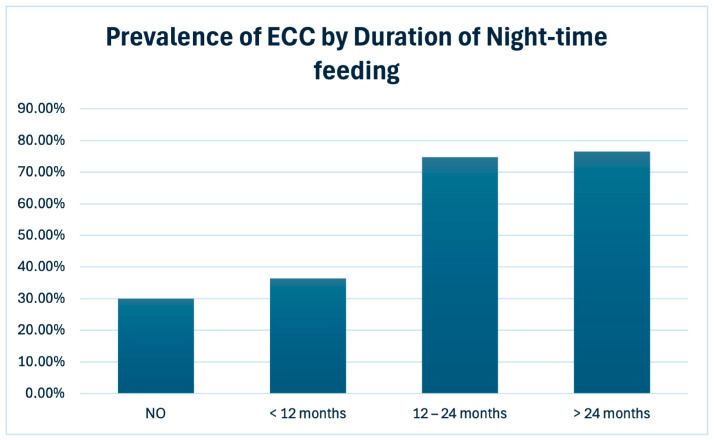
Prevalence of Early Childhood Caries (ECC) according to the duration of night-time feeding. A progressive increase in ECC prevalence is observed with increasing duration of night-time feeding, from absence of night-time feeding to durations exceeding 24 months.

The Chi-square test demonstrated highly significant differences across duration categories (χ^2^ = 22.73; df = 3; *p* < 0.001), with a progressive increase in ECC prevalence as feeding duration increased. Children who were fed at night for more than 24 months exhibited the highest prevalence of ECC, confirming exposure duration as a critical factor (Figure 4).

### 3.3. Type of Night-Time Feeding

A statistical analysis was conducted to evaluate the association between three categories of night-time feeding (NO: no night-time feeding; NATURAL: breast milk; OTHER: formula milk, cow’s milk, and milk with biscuits) and the presence of Early Childhood Caries (ECC) (Table 3). The recategorization was performed based on the original dataset after excluding cases with missing diagnosis or undefined feeding type.

A 3 × 2 contingency analysis was performed comparing the three feeding categories with the outcome (ECC vs. no ECC). The global Chi-square test showed a statistically significant difference among groups (χ^2^ = 14.88; *p* < 0.001), indicating an overall association between the type of night-time feeding and the presence of ECC.

Additional pairwise comparisons were performed using Fisher’s exact test (2 × 2 contrasts) to assess the risk associated with each feeding category relative to the others. The NO group showed an odds ratio of 0.17 (*p* = 0.00057), indicating a significantly lower risk of developing ECC compared with children receiving any form of night-time feeding. The NATURAL feeding group showed an odds ratio of 1.16 (*p* = 0.657), with no statistically significant association. The OTHER feeding group showed an odds ratio of 1.72 (*p* = 0.097), suggesting a trend toward increased ECC risk, although this result did not reach statistical significance.

### 3.4. Interaction Between Duration and Type of Night-Time Feeding

A multivariable logistic regression analysis was performed to simultaneously evaluate the effects of night-time feeding duration and type of feeding on the risk of developing Early Childhood Caries (ECC). The independent variables included: (i) duration of night-time feeding (DUR), categorized into four levels (0 = no night-time feeding; 1 = <12 months; 2 = 12–24 months; 3 = >24 months); and (ii) type of night-time feeding (NATURAL, OTHER, NO), with NATURAL used as the reference category. The outcome variable was the presence of ECC (0 = no ECC; 1 = ECC) (Table 4 and Table 5). Only children with complete data for diagnosis, type, and duration of night-time feeding were included in the analysis.

Night-time feeding duration showed a significant association with ECC risk. Each incremental increase in duration category was associated with an approximately 2.35-fold increase in the odds of ECC (OR ≈ 2.35; *p* < 0.001), indicating a progressive increase in risk with longer exposure. Regarding feeding type, no statistically significant difference was observed between OTHER feeding (including formula milk, cow’s milk, and milk with biscuits) and NATURAL feeding (breast milk) (OR ≈ 1.4; *p* = 0.33), suggesting that the type of feeding does not significantly influence ECC risk when duration is taken into account. In contrast, the absence of night-time feeding was associated with a significantly reduced risk of ECC (OR ≈ 0.25; *p* = 0.002), corresponding to an approximate 75% reduction in risk compared with children receiving night-time feeding. Overall, these findings indicate that the duration of night-time feeding is the primary determinant of ECC risk, whereas the type of feeding plays a secondary and non-significant role when both variables are considered simultaneously. The absence of night-time feeding, on the other hand, emerges as a relevant protective factor.

### 3.5. Night-Time Feeding and Sugar-Rich Diet

A statistical analysis using Fisher’s exact test was performed to evaluate whether the presence of a sugar-rich diet further increases the risk of Early Childhood Caries (ECC) in children undergoing night-time feeding. To improve statistical power, the duration of night-time feeding was dichotomized into two categories: <12 months and ≥12 months (including both 12–24 months and >24 months) (Table 6 and Table 7). Only children with explicitly reported data on sugar intake (“yes” or “no”) were included in the analysis, while cases with missing or undefined information were excluded.

For each duration group, a 2 × 2 contingency analysis (sugar-rich diet: yes/no × ECC: yes/no) was performed. In children with night-time feeding duration < 12 months, no significant association was observed between a sugar-rich diet and ECC (OR = 0.75; *p* = 1.00), indicating no detectable effect of sugar intake in this subgroup. In contrast, among children with night-time feeding duration ≥ 12 months, a sugar-rich diet was associated with a markedly increased risk of ECC (OR = 4.25; *p* < 0.001). Specifically, children exposed to both prolonged night-time feeding and a sugar-rich diet exhibited more than a fourfold higher risk of ECC compared with those not consuming high levels of sugar. Overall, these findings indicate that the effect of a sugar-rich diet on ECC risk is strongly dependent on the duration of night-time feeding. While no significant increase in risk is observed before 12 months, the combination of prolonged night-time feeding and high sugar intake represents a powerful and clinically relevant risk factor for ECC.

### 3.6. Oral Hygiene Habits

The association between the type of toothbrush used and the presence of Early Childhood Caries (ECC) was analyzed after excluding all cases with missing data (“ND”). Children were classified into two groups: manual toothbrush and electric toothbrush. (Table 8). The electric toothbrush category also included children reported as using both manual and electric toothbrushes.

A Chi-square (χ^2^) test was performed to assess whether the difference between the two groups was statistically significant. The analysis showed a significant association between toothbrush type and ECC (χ^2^ = 6.72; df = 1; *p* = 0.0095). The use of a manual toothbrush was associated with a higher prevalence of ECC, whereas the use of an electric toothbrush was associated with a lower risk of ECC.

### 3.7. Number of Daily Meals

The association between the number of daily meals and the presence of Early Childhood Caries (ECC) was analyzed. Children were categorized into two groups: ≤5 meals per day and >5 meals per day (Table 9). Only children with complete data for both meal frequency and ECC diagnosis were included in the analysis.

A Chi-square (χ^2^) test was performed to assess the presence of a statistically significant association. The analysis showed a significant association between meal frequency and ECC (χ^2^ = 5.00; df = 1; *p* = 0.025). Children consuming more than five meals per day exhibited a markedly higher prevalence of ECC compared with those consuming five or fewer meals per day.

### 3.8. Fluoridated Toothpaste

The association between the use of fluoridated toothpaste and the presence of Early Childhood Caries (ECC) was analyzed. Only children with complete data for both dental diagnosis and toothpaste use were included. A Chi-square (χ^2^) test was performed to evaluate the presence of a statistically significant association. No significant association was found between fluoridated toothpaste use and ECC (χ^2^ = 0.0062; df = 1; *p* = 0.937). The prevalence of ECC was similar in children using fluoridated toothpaste and those not using it (approximately 64% and 67%, respectively). These findings do not provide statistical evidence that the reported use of fluoridated toothpaste influenced ECC prevalence in the analyzed sample.

### 3.9. Toothbrushing Frequency

The association between toothbrushing frequency and ECC was analyzed. The variable “toothbrushing frequency” was recoded into two groups: ≤1 time per day (including responses such as never, monthly, weekly, and once daily) and >1 time per day (Table 10).

A Chi-square (χ^2^) test was applied to assess the presence of a statistically significant association. No significant association was observed between toothbrushing frequency and ECC (χ^2^ = 0.00; df = 1; *p* = 1.00). The prevalence of ECC was nearly identical between the two groups, indicating that brushing frequency alone was not associated with ECC in this sample.

### 3.10. Age at Initiation of Toothbrushing

Age at initiation of toothbrushing showed a clear dose–response relationship with ECC presence. Children were categorized into four groups: before 1 year of age, 1–2 years, 2–3 years, and >3 years. Responses indicating “never” were included in the >3 years category (Table 11).

A Chi-square (χ^2^) test revealed a statistically significant association between age at initiation of toothbrushing and ECC (χ^2^ = 11.16; df = 3; *p* = 0.0109).

A progressive increase in ECC prevalence was observed with delayed initiation of toothbrushing: 41.7% in children who started before 1 year of age, 65.8% in those starting between 1–2 years, 73.7% between 2–3 years, and 78.7% in those starting after 3 years.

These findings suggest that early initiation of toothbrushing—particularly within the first year of life—represents an important protective factor against ECC, whereas delayed initiation is associated with a significantly increased risk.

### 3.11. Multivariable Logistic Regression: Factors Associated with ECC Presence

**Figure 5:**
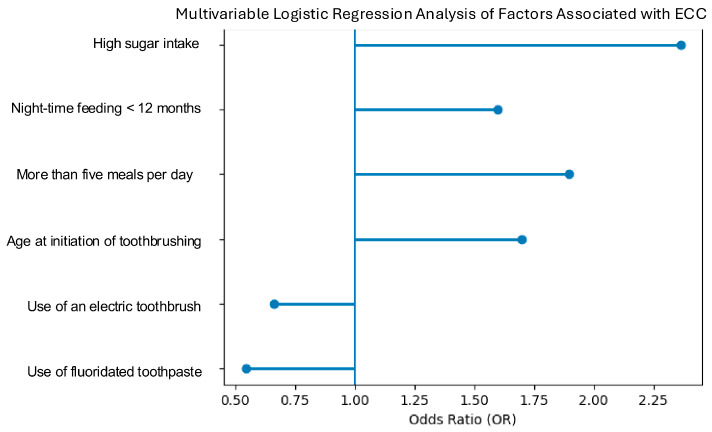
Forest plot of multivariable logistic regression showing associations between key dietary and behavioral factors and Early Childhood Caries (ECC). Odds ratios (OR) are shown. OR > 1 indicates increased risk, whereas OR < 1 indicates a protective effect.

To further validate the previous findings, a multivariable logistic regression analysis was performed using ECC presence as the outcome variable (1 = ECC/S-ECC; 0 = no ECC). Only subjects with complete data for all variables were included in the model (63 with ECC/S-ECC and 27 without ECC). (Table 12 and Figure 5).

A detailed presentation of the multivariable logistic regression results is reported in Table 13, including odds ratios (OR), 95% confidence intervals (CI), and *p*-values for all predictors included in the model. The overall model was statistically significant (likelihood ratio test, *p* = 0.0357), with a McFadden pseudo-R^2^ of 0.176.

In the multivariable analysis, none of the individual predictors reached statistical significance at the conventional threshold (*p* < 0.05). However, several variables showed trends toward association, including high-sugar diet (OR = 2.95, *p* = 0.077) and delayed initiation of toothbrushing after 3 years (OR = 4.59, *p* = 0.099). Use of an electric toothbrush showed a potential protective effect (OR = 0.37, *p* = 0.089), although this did not reach statistical significance.

The model coefficients (β) indicated that a sugar-rich diet (β = 0.86) and night-time feeding duration > 12 months (β = 0.47) were among the strongest risk factors for ECC. A higher number of daily meals (>5 per day) was also associated with increased risk (β = 0.64).

Regarding oral hygiene variables, electric toothbrush use (β = −0.41) and fluoridated toothpaste use (β = −0.61) were associated with a reduced probability of ECC, suggesting a protective effect. In contrast, toothbrushing frequency > 1 time/day did not show a meaningful association (β = 0.10), indicating that frequency alone may not reflect effective oral hygiene.

Age at initiation of toothbrushing (β = 0.53) showed a clear positive association with ECC risk, with progressively higher risk observed as brushing initiation was delayed. Children who began toothbrushing after 3 years of age exhibited substantially higher odds of ECC compared with those who started within the first year of life, even after adjusting for dietary habits, night-time feeding, and fluoride use.

The presence of night-time feeding as a binary variable (yes/no) did not show an independent effect once feeding duration was included in the model, confirming that duration is a more informative determinant.

Overall, the model indicates that dietary factors—particularly sugar intake, meal frequency, and prolonged night-time feeding—appear to be the main contributing factors of ECC risk in the analyzed sample. Protective factors are primarily related to the quality of oral hygiene, including the use of an electric toothbrush and fluoridated toothpaste. Conversely, self-reported toothbrushing frequency does not appear to be a reliable predictor, likely due to variability in brushing effectiveness and potential reporting bias (Table 14).

## 4. Discussion

The results of the present study suggest that the development of Early Childhood Caries (ECC) is the result of a complex interaction between dietary and behavioral factors, whereas oral hygiene–related variables, when considered in isolation, show a less pronounced impact in the analyzed sample. In particular, the central role of night-time feeding duration appears to be a key contributing factor.

Children who do not receive night-time feeding represent the group with the lowest prevalence of ECC and may therefore be considered the reference baseline. In contrast, continuation of night-time feeding beyond 12 months of age is associated with a progressive increase in ECC prevalence, exceeding 75% among children fed at night between 12–24 months and beyond 24 months. This risk gradient suggests that the duration of night-time feeding, rather than its mere presence, is the key factor driving the increased risk of ECC.

This observation is consistent with the physiology of the oral cavity during sleep, which is characterized by reduced salivary flow, decreased buffering capacity, and absence of active cleansing mechanisms [13,14]. Under these conditions, prolonged retention of fermentable carbohydrates on tooth surfaces promotes a persistent acidogenic environment, thereby increasing susceptibility to enamel demineralization [15].

Analysis of the type of night-time feeding shows that children who do not receive any night-time feeding are significantly protected compared with all other groups. However, when controlling for feeding duration, the type of milk (breast milk vs. other types) loses statistical significance. This finding suggests that ECC risk is not primarily determined by the composition of the feed, but rather by the mode and duration of exposure, confirming that the temporal dimension is more informative than the qualitative one.

Although night-time breastfeeding does not emerge as an independent risk factor compared with other feeding types, it is associated with a high prevalence of ECC when prolonged over time. This is compatible with the biochemical composition of human milk, which contains lactose, a fermentable carbohydrate whose concentration does not decrease with extended breastfeeding [16]. Under normal daytime conditions, its cariogenic potential is mitigated by adequate salivary clearance and oral hygiene; however, during night-time hours, the absence of toothbrushing and reduced salivary secretion render the oral cavity particularly vulnerable. Similarly, infant formulas and milk-based preparations with added biscuits or simple sugars create even more favorable conditions for acidogenic bacterial proliferation.

A particularly relevant finding concerns the interaction between night-time feeding and a high-sugar diet. Among children with night-time feeding limited to less than 12 months, sugar intake does not appear to significantly modify ECC prevalence. In contrast, in children with night-time feeding extending beyond 12 months, the concomitant presence of a high-sugar diet is associated with a higher risk, with an odds ratio greater than 4. This finding highlights a synergistic effect between prolonged nocturnal exposure and frequent sugar consumption, defining a high-caries-risk scenario.

The number of daily meals is also confirmed as a significant determinant: children consuming more than five meals per day show a significantly higher prevalence of ECC. This result is consistent with the pathophysiological model of caries described by the Stephan curve [17], according to which each eating episode induces a drop in oral pH, and the frequency of acidogenic events represents an independent risk factor, regardless of total sugar quantity.

With regard to oral hygiene habits, use of an electric toothbrush was associated with a lower prevalence of ECC, suggesting a possible protective effect, likely related to greater mechanical efficacy in plaque removal in an age group characterized by limited manual dexterity and variable parental supervision.

No statistically significant association was observed between the use of fluoridated toothpaste and ECC, either in the univariate analysis or in the multivariable model. However, the adjusted odds ratio below 1 suggests a possible trend toward a protective effect.

This lack of statistical significance may be related to the relatively small sample size available for multivariable analysis, resulting in limited statistical power and wide confidence intervals. Nevertheless, the direction of the observed effect is consistent with existing evidence supporting the protective role of fluoride against dental caries.

Age at initiation of toothbrushing is confirmed as another key element: early introduction of oral hygiene, ideally coinciding with eruption of the first teeth, is associated with a significantly lower risk of ECC, whereas postponement beyond the first years of life is associated with a progressive increase in risk. In contrast, reported toothbrushing frequency does not emerge as a significant predictor, suggesting that this self-reported variable may not adequately capture quality, technique, and level of supervision.

Overall, these results indicate that ECC prevention requires a multifactorial approach in which primary attention should be directed toward dietary behaviors, particularly management of night-time feeding beyond the first year of life, reduction in sugar exposure, and limitation of meal frequency. Oral hygiene practices retain a fundamental protective role but are not sufficient, on their own, to counterbalance high-risk dietary habits.

Several limitations should be considered when interpreting the findings of this study. The retrospective, single-center design may have introduced selection bias, as the study population comprised children referred for a first dental visit at a hospital-based pediatric dentistry unit, potentially reflecting a higher-risk subgroup rather than the general pediatric population.

Exposure variables, including dietary habits, night-time feeding practices, and oral hygiene behaviors, were based on parental reports and are therefore subject to recall bias and possible social desirability bias, which may have affected the accuracy of the collected information.

Although multivariable analyses were performed, residual confounding cannot be ruled out. Potentially relevant factors—such as socioeconomic status, parental education, access to dental care, and microbiological determinants—were not systematically captured and could not be included in the model.

Finally, due to the observational nature of the study, the observed associations should be interpreted with caution, as causality cannot be inferred.

## 5. Conclusions

The present study highlights that night-time feeding prolonged beyond 12 months of age, particularly when combined with a high-sugar diet and a high frequency of daily meals, represents a primary and modifiable risk factor for the development of Early Childhood Caries. Exposure duration emerges as the key determinant, outweighing the type of food consumed. Early introduction of oral hygiene practices, use of fluoridated toothpaste, and effective plaque-removal tools constitute fundamental preventive strategies; however, these measures must be integrated within a broader educational intervention.

These findings emphasize the need for clear, consistent, and shared preventive messages among pediatricians, dentists, midwives, and breastfeeding counselors, in order to guide families toward informed management of night-time feeding and oral health during early childhood. A multidisciplinary and early-life approach represents the most effective strategy to reduce ECC incidence and to promote a lasting culture of prevention.

## Figures and Tables

**Table 1:** Definition of Early Childhood Caries (ECC) and Severe Early Childhood Caries (S-ECC) according to age, adapted from the American Academy of Pediatric Dentistry (AAPD) criteria [1] based on previously proposed standardized definitions [7].

Age (Months)	Early Childhood Caries (ECC)	Severe Early Childhood Caries (S-ECC)
<12	≥1 decayed (non-cavitated or cavitated), missing (due to caries), or filled surface in any primary tooth	Any sign of caries on a smooth surface
12–23	≥1 decayed, missing (due to caries), or filled surface.	≥1 smooth-surface decayed, missing, or filled surface
24–35	≥1 decayed, missing (due to caries), or filled surface	≥1 smooth-surface decayed, missing, or filled surface
36–47	≥1 decayed, missing (due to caries), or filled surface	≥1 smooth-surface cavitated, filled, or missing (due to caries) surface in primary maxillary anterior teeth or dmfs ≥ 4
48–59	≥1 decayed, missing (due to caries), or filled surface	Criteria in primary maxillary anterior teeth or dmfs ≥ 5
60–71	≥1 decayed, missing (due to caries), or filled surface	Criteria in primary maxillary anterior teeth or dmfs ≥ 6

**Table 2:** Distribution of Early Childhood Caries (ECC) according to the duration of night-time feeding.

Duration of Night-Time Feeding	NO ECC	ECC
0 = NO	14	6
1 = <12 months	7	4
2 = 12–24 months	19	56
3 = >24 months	19	62

**Table 3:** Distribution of Early Childhood Caries (ECC) according to the type of night-time feeding.

Type of Night-Time Feeding	NO ECC	ECC
NO	14	6
NATURAL	31	70
OTHER	22	64

**Table 4:** Estimated cumulative odds ratio and corresponding interpretation of ECC risk according to the duration of night-time feeding.

Duration	Cumulative Odds Ratio (Approximate)	Interpretation
NO night-time feeding	1.0 (baseline)	Minimal risk
<12 months	≈2.3×	Approximately doubled risk
12–24 months	≈(2.3^2^) ≈ 5.4×	Markedly increased risk
>24 months	≈(2.3^3^) ≈ 12.5×	Very high risk

**Table 5:** Estimated ECC risk according to type and duration of night-time feeding.

Type of Night-Time Feeding	Duration (Code)	Duration (Months)	ECC Risk
NO	0	-	0.30 (30%)
NATURAL	1	<12 months	0.33 (33.3%)
NATURAL	2	12–24 months	0.74 (74.1%)
NATURAL	3	>24 months	0.74 (73.5%)
OTHER	1	<12 months	0.50 (50%)
OTHER	2	12–24 months	0.76 (76.2%)

**Table 6:** Association between sugar-rich diet and ECC according to duration of night-time feeding (<12 months).

Sugar-Rich Diet < 12 Months	NO ECC	ECC
NO sugar	3	2
YES sugar	4	2

**Table 7:** Association between sugar-rich diet and ECC according to duration of night-time feeding (≥12 months).

Sugar-Rich Diet > 12 Months	NO ECC	ECC
NO sugar	17	19
YES sugar	20	95

**Table 8:** Distribution of Early Childhood Caries (ECC) according to the type of toothbrush.

Type of Toothbrush	NO ECC	ECC
ELECTRIC	22	20
MANUAL	45	108

**Table 9:** Distribution of Early Childhood Caries (ECC) according to the number of daily meals.

Number of Daily Meals	NO ECC	ECC
≤5 meals	65	103
>5 meals	6	29

**Table 10:** Distribution of Early Childhood Caries (ECC) according to the toothbrushing frequency.

Toothbrushing Frequency	NO ECC	ECC
≤1	22	43
>1	45	87

**Table 11:** Distribution of Early Childhood Caries (ECC) according to the age at initiation of toothbrushing.

Age at Initiation of Toothbrushing	ECC/S-ECC	NO ECC
Before 1 year of age	10	14
1–2 years	25	13
2–3 years	42	15
>3 years	37	10

**Table 12:** Variables included in the multivariable logistic regression model.

Variable	Description
Toothbrushing frequency (FREQ_HIGH)	(1 = >1 time/day; 0 = ≤1 time/day;)
Electric toothbrush use (ELEC)	1 = yes; 0 = manual toothbrush
Fluoridated toothpaste use (DENT)	(1 = yes; 0 = no)
High-sugar diet (ZUC)	1 = yes; 0 = no
Night-time feeding duration > 12 months (DUR12)	1 = yes; 0 = ≤12 months
Presence of night-time feeding (NF)	1 = yes; 0 = no
Number of daily meals > 5 (MEAL_HIGH)	1 = yes; 0 = ≤5
Age at initiation of toothbrushing (AGE_BRUSH)	<1, 1–2, 2–3, >3 years

**Table 13:** Multivariable logistic regression analysis of factors associated with Early Childhood Caries (ECC). Odds ratios (OR), 95% confidence intervals (CI), and *p*-values are reported for each predictor included in the model.

Predictor	OR	95% CI	*p*-Value
Toothbrushing > 1/day	1.55	0.46–5.20	0.476
Electric toothbrush	0.37	0.12–1.16	0.089
Fluoridated toothpaste	0.74	0.16–3.35	0.698
High-sugar diet	2.95	0.89–9.78	0.077
Night-time feeding	2.62	0.44–15.61	0.289
Night-time feeding > 12 months	1.98	0.53–7.42	0.309
>5 meals/day	2.06	0.38–11.25	0.402
Brushing start 1–2 years	3.70	0.72–19.18	0.119
Brushing start 2–3 years	3.07	0.55–17.17	0.202
Brushing start >3 years	4.59	0.75–28.06	0.099

**Table 14:** Clinical interpretation of the multivariable logistic regression findings for ECC.

Factor	Direction of Association with ECC	Clinical Interpretation
High-sugar diet	Increased risk	Children with a high-sugar diet have a higher probability of ECC
Night-time feeding > 12 months	Increased risk	Prolonged night-time feeding increases ECC risk
>5 meals per day	Increased risk	Higher daily meal frequency increases ECC risk
Age at initiation of toothbrushing	Increased risk with delay	Later start of toothbrushing increases ECC risk
Use of electric toothbrush	Reduced risk	Electric toothbrush use is protective
Use of fluoridated toothpaste	Reduced risk	Fluoridated toothpaste use is protective
Toothbrushing ≥ 2 times/day	No significant association	Frequency alone was not associated with ECC
Presence of night-time feeding (yes/no)	No significant association	Duration matters more than presence

## Data Availability

The data presented in this study are available on request from the corresponding author. The data are not publicly available due to privacy restrictions.

## Supplementary Materials

The following supporting information can be downloaded at: https://www.mdpi.com/article/10.3390/children13040489/s1, Supplementary File S1: Standardized parent-completed questionnaire used at the first dental visit. Table S2: Study categories and variables.

## Abbreviations

The following abbreviations are used in this manuscript:

ECC	Early Childhood Caries
S-ECC	Severe Early Childhood Caries
AAPD	American Academy of Pediatric Dentistry
OR	Odds Ratio
