# Supplementary material for: The Role of Dietary Habits, Night-Time Feeding and Oral Hygiene in Early Childhood Caries: A Retrospective Observational Study in 248 Children from Southern Italy
Source: Children (Basel). 2026 Mar 31;13(4):489. doi: 10.3390/children13040489 (PMC13115513; doi:10.3390/children13040489)
Supplement: Supplementary file 1 [file children-13-00489-s001.zip › children-4199974-supplementary.pdf]

SUPPLEMENTARY MATERIAL

S1: Standardized parent-completed questionnaire used at the first dental visit

ANAMNESI FISIOLOGICA

La **gravidanza** ha avuto un normale decorso? SI NO
Se NO specificare
____
____

Parto:
- ☐ naturale
- ☐ taglio cesareo
- ☐ a termine
- ☐ prematuro (settimane di gestazione _______________)

Allattamento:
- ☐ naturale fino a ________ mesi
- ☐ artificiale fino a ________ mesi

Tappe di sviluppo (prensione, stazione eretta, linguaggio, gioco, ecc.):
- ☐ precoci
- ☐ nella norma
- ☐ tardive

Note:____
____

Alvo
- ☐ normale
- ☐ stitico
- ☐ diarroico

Diuresi
- ☐ Normale
- ☐ oliguria
- ☐ poliuria

Inserimento scolastico:
ha frequentato l'asilo **nido**? SI NO
a che età?________________________
ha frequentato la scuola **materna**? SI NO
a che etá?________________________

Attualmente frequenta:
Scuola____
Classe____
Rendimento____

ANAMNESI PATOLOGICA REMOTA

Suo/a figlio/a ha avuto **problemi alla nascita**? SI NO
se SI specificare____
____

Suo/a figlio/a ha eseguito le **vaccinazioni**? SI NO
____

Quali delle seguenti **malattie tipiche dell'infanzia** ha avuto suo/a figlio/a

- ☐ morbillo età ________
- ☐ parotite età ________
- ☐ rosolia età ________
- ☐ varicella età ________
- ☐ pertosse età ________
- ☐ scarlattina età ________
- ☐ mano-piede-bocca età ________
- ☐ quinta malattia età ________
- ☐ sesta malattia età ________

Suo/a figlio/a soffre/ha sofferto in passato delle seguenti **malattie**?

- **malattie autoimmunitarie** SI NO
Se SÌ specificare____

- **sindromi genetiche** SI NO
Se SÌ specificare____

- **malattie gastriche e/o dell'apparato digerente** SI NO
Se SÌ specificare____

- **malattie nervose** SI NO
Se SÌ specificare____

- **epilessia, convulsioni** SI NO
Se SÌ specificare____

- **malattie cardiache** SI NO
Se SÌ specificare____

- **malattie polmonari (bronchite, asma...)** SI NO
Se SÌ specificare____

- **malattie renali** SI NO
Se SÌ specificare____

- **epatiti o altre malattie del fegato** SI NO
Se SÌ specificare____

- **malattie ormonali** SI NO
Se SÌ specificare____

- **malattie metaboliche** SI NO
Se SÌ specificare____

- **diabete** SI NO
Se SÌ specificare____

- **patologie oncologiche** SI NO
Se SÌ specificare____

- **malattie del sangue/problemi di sanguinamento** SI NO
Se SÌ specificare____

- **anemia**? SI NO
Se SÌ specificare____

- **allergie**? SI NO
Se SÌ specificare____

- **celiachia**? SI NO
Se SÌ specificare____

- **altro**? SI NO
Se SÌ specificare____

Ricoveri: SI NO
Se SÌ specificare____
____
____

Interventi chirurgici: SI NO
Se SÌ specificare____
____
____

Traumi: SI NO
Se SÌ specificare____
____
____

ANAMNESI FARMACOLOGICA

Suo/a figlio/a sta eseguendo o ha eseguito in passato:

- ☐ radioterapia testa-collo
- ☐ chemioterapia per patologie oncologiche
- ☐ terapie immunosoppressive

Suo/a figlio/a assume o ha assunto **per periodi prolungati** uno o più dei seguenti farmaci:

- ☐ sciroppi
- ☐ antibiotici
- ☐ aerosol a base di cortisone
- ☐ antiepilettici
- ☐ antistaminici
- ☐ insulina o antidiabetici orali
- ☐ terapie omeopatiche con granuli
- ☐ altro: ______________________

Ricorda il nome dei farmaci che Suo/a figlio/a ha assunto in passato?

____

____

____

ANAMNESI PATOLOGICA PROSSIMA

Stato di salute **attuale**:

____

Patologia **in corso**:

____

Terapia **in corso** (farmaco e dosaggio)

____

____

____

____

____

Suo/a figlio/a é in cura presso:
Ospedale/Istituto _____________________________ Reparto _________________
Ospedale/Istituto _____________________________ Reparto _________________

Suo/a figlio/a é seguito da:
Dott. __________________ specializzazione PEDIATRIA tel ________________
Dott. __________________ specializzazione ____________ tel ________________
Dott. __________________ specializzazione ____________ tel ________________

QUESTIONARIO ALIMENTAZIONE

Quante volte mangia suo/a figlio/a al giorno?

☐ due volte (pranzo, cena, non fa merende)
☐ tre volte (colazione, pranzo, cena)
☐ quattro volte (colazione, pranzo, cena, una merenda)
☐ cinque volte (colazione, pranzo, cena, due merende)
☐ sei o sette volte (supera le due merende)
☐ più di sette volte (molti fuori pasto)

Alimenti sgraditi:____

Intolleranze: ____

Alimentazione serale/notturna (anche in passato) SI NO

☐ latte materno
☐ latte con biscotti
☐ camomilla
☐ tisane
☐ altro: ________________________________

Quante volte suo/a figlio/a mangia o beve i cibi e le bevande indicati, anche in piccola quantità?

gomme da masticare	☐ spesso	☐ raramente	☐ mai
caramelle	☐ spesso	☐ raramente	☐ mai
lecca-lecca	☐ spesso	☐ raramente	☐ mai
latte con zucchero	☐ spesso	☐ raramente	☐ mai
latte e cacao	☐ spesso	☐ raramente	☐ mai
tè con zucchero	☐ spesso	☐ raramente	☐ mai
merendine	☐ spesso	☐ raramente	☐ mai
biscotti	☐ spesso	☐ raramente	☐ mai
frutta fresca	☐ spesso	☐ raramente	☐ mai
succhi di frutta	☐ spesso	☐ raramente	☐ mai
coca cola	☐ spesso	☐ raramente	☐ mai
bibite gassate	☐ spesso	☐ raramente	☐ mai
miele	☐ spesso	☐ raramente	☐ mai
cioccolata	☐ spesso	☐ raramente	☐ mai

ANAMNESI ODONTOIATRICA

Stato di salute orale di genitori e fratelli:

La **madre** ha una buona salute orale?	SI	NO
Ha eseguito molte cure dentali?	SI	NO
Ha eseguito una terapia ortodontica?	SI	NO
Il **padre** ha una buona salute orale?	SI	NO
Ha eseguito molte cure dentali?	SI	NO
Ha eseguito una terapia ortodontica?	SI	NO
I **fratelli** hanno una buona salute orale?	SI	NO
Hanno eseguito molte cure dentali?	SI	NO
Hanno eseguito una terapia ortodontica?	SI	NO

A che età suo/a figlio/a ha cominciato a spazzolare i denti? ___________________

Suo/a figlio/a lava i denti da solo/a? SI NO

Con quale frequenza suo/a figlio/a pulisce i denti?

- ☐ mai
- ☐ qualche volta al mese (2-3)
- ☐ qualche volta a settimana (2-6)
- ☐ una volta al giorno
- ☐ due volte al giorno
- ☐ tre o piú volte al giorno

Cosa usa per pulire denti e gengive?

- ☐ spazzolino elettrico
- ☐ spazzolino manuale
- ☐ filo interdentale
- ☐ scovolino
- ☐ nettalingua
- ☐ collutorio
- ☐ dentifricio con fluoro
- ☐ dentifricio senza fluoro
- ☐ altro: __________________

Suo/a figlio/a assume fluoro in altro modo, oltre a dentifricio e collutorio?

- ☐ compresse o gocce
- ☐ gel al fluoro ad uso domiciliare
- ☐ gel o vernici ad uso ambulatoriale

Quante volte suo/a figlio/a è andato/a dal dentista negli ultimi 12 mesi, incluso l'ortodontista:

- ☐ mai
- ☐ una volta
- ☐ due volte
- ☐ tre volte
- ☐ più di tre volte
- ☐ non lo so

Suo/a figlio/a é già stato da un dentista? SI NO

Se SI per quale motivo?

☐	per visite di controllo	☐	per dolore e gonfiore
☐	per carie	☐	per denti storti
☐	per trauma	☐	altro:______________________

Quali trattamenti ha eseguito?

☐	nessuno	☐	otturazioni
☐	solo visita	☐	estrazioni
☐	igiene e profilassi	☐	altro: ______________________

Suo/a figlio/a é stato mai sottoposto ad anestesia locale? SI NO

Ha mai avuto problemi per un trattamento odontoiatrico? SI NO
Se SÌ specificare____
____

Suo/a figlio/a ha/ha avuto qualcuna delle seguenti abitudini?

☐ utilizzare il ciuccio
☐ succhiare dita, labbra, guance
☐ rosicchiare gli oggetti
☐ mordicchiarsi le labbra
☐ rosicchiare le unghie

Suo/a figlio/a ha/ha avuto problemi di respirazione? SI NO

☐ ha difficoltà a tenere le labbra chiuse
☐ si ammala spesso
☐ ha le tonsille grosse
☐ dorme a bocca aperta
☐ bagna il cuscino di saliva
☐ russa
☐ va in apnea mentre dorme
☐ si sveglia con bocca secca e alito cattivo

Comportamento:

☐ sereno/collaborativo ☐ diffidente/pauroso

ANAMNESI FAMILIARE

La **MADRE** gode di buona salute? SI NO
Se NO specificare____
____

Il **PADRE** gode di buona salute? SI NO
Se NO specificare____
____

FRATELLI E SORELLE:

1- Nome______________________________________data di nascita_______________
gode di buona salute? SI NO
Se NO specificare
____

2- Nome______________________________________data di nascita_______________
gode di buona salute? SI NO
Se NO specificare
____

3- Nome______________________________________data di nascita_______________
gode di buona salute? SI NO
Se NO specificare
____

Specificare eventuali patologie croniche a carattere familiare anche di **ALTRI FAMILIARI**: nonni, zii, ecc.

- ☐ diabete ____
- ☐ ipertensione ____
- ☐ patologie cardiache ____
- ☐ allergie ____
- ☐ tumori ____
- ☐ celiachia ____
- ☐ anomalie dentarie ____
- ☐ sindromi genetiche ____
- ☐ altro ____

S2: Study categories and variables

Categoria	Variabile	Numero	Percentuale
SESSO	M	145	58.47%
	F	103	41.53%
DIAGNOSI	ECC/S-ECC	155	62.50%
	NO ECC-ALTRO	89	35.89%
	ND	4	1.61%
ETÀ	5–6 anni	111	44.76%
	3–4 anni	95	38.31%
	1–2 anni	42	16.94%
NUMERO PASTI	5	92	37.10%
	4	57	22.98%
	ND	44	17.74%
	6	25	10.08%
	3	17	6.85%
	7	9	3.63%
	2	4	1.61%
ALIM. NOTTURNA	Naturale	98	39.52%
	Formula	46	18.55%
	No	20	8.06%
	ND	18	7.26%
	Naturale + Formula	5	2.02%
	Naturale + Latte + Biscotti	7	2.82%
	Latte	3	1.21%
	Latte + Biscotti	20	8.06%
	Altro	9	3.63%
DURATA ALIM NOT	ND	59	23.79%
	No	20	8.06%
	≤12 mesi	27	10.89%
	12–24 mesi	60	24.19%
	>24 mesi	82	33.06%

DIETA ZUCCHERI	Sì	158	63.71%
	No	51	20.56%
	ND	39	15.73%
INIZIO SPAZZOL.	ND	62	25.00%
	Entro 1 anno	63	25.40%
	Entro 2 anni	67	27.02%
	Entro 3 anni	33	13.31%
	Entro 4 anni	11	4.44%
	Entro 5 anni o oltre	6	2.42%
	Mai	5	2.02%
FREQ. SPAZZ.	2 volte/die	116	46.77%
	1 volta/die	58	23.39%
	ND	43	17.34%
	3 volte/die	18	7.26%
	Settimanale	6	2.42%
	Mai	5	2.02%
	Mensile	2	0.81%
TIPO SPAZZOLINO	Manuale	141	56.85%
	ND	48	19.35%
	Elettrico	42	16.94%
	Elettrico + Manuale	14	5.65%
	No	3	1.21%
DENTIFRICIO FLUORO	ND	120	48.39%
	Sì	103	41.53%
	No	25	10.08%
